# Geographical inequalities in lung cancer management and survival in South East England: evidence of variation in access to oncology services?

**DOI:** 10.1038/sj.bjc.6602247

**Published:** 2004-11-09

**Authors:** R H Jack, M C Gulliford, J Ferguson, H Møller

**Correction to**: *British Journal of Cancer* (2003) **88**, 1025–1031. doi: 10.1038/sj.bjc.6600831

The authors have recently notified us of an error in the above paper. It had come to their attention that, due to a coding error, a number of patients who received chemotherapy or radiotherapy were misclassified as having no treatment. The proportions of patients receiving any chemotherapy, radiotherapy and any active treatment were too low as reported. The median percentage and range of patients receiving different treatments within the health authorities are shown below in the revised Table 3

**Table 3:** (revised) Median percentage and range of lung cancer patients with each treatment by health authority, 1995–1999

	**Med %**	**Range**
None registered^a^	38	32–55
Chemotherapy only (Chem)	10	6–17
Radiotherapy only (RT)	32	25–44
Non-investigative surgery only (S)	6	4–10
Chem+RT	9	5–14
S+Chem	0	0–1
S+RT	1	0–3
S+Chem+RT	0	0–1
		
Investigative surgery only	20	15–40
		
Any Chem	20	14–29
Any RT	42	31–54
Any S	7	5–12
Any treatment^b^	62	45–68
		
First hospital visited a radiotherapy centre	27	8–79

a Includes patients who had investigative surgery only.

b Any treatment=any chemomerapy, radiotherapy or non-investigative surgery.

.

In the revised analyses, there was still evidence of health authority level variation in treatment and survival similar to that shown in Figure 1. The associations of area and health service characteristics are shown in the revised Table 5

**Table 5:** (revised) Area and organisational characteristics associated with treatment and survival

	**OR**	**95% CI**	***P*-value**
*Any treatment*			
First hospital visited a radiotherapy centre	1.84	1.46–2.32	<0.0001
Deprivation (Townsend score)	0.98	0.96–0.99	0.0091
Age standardised incidence (per 100 000)	1.00	0.99–1.01	0.9128
			
*Any chemotherapy*			
First hospital visited a radiotherapy centre	1.24	0.98–1.55	0.0699
Deprivation (Townsend score)	0.96	0.94–0.98	0.0001
Age standardised incidence (per 100 000)	1.02	1.01–103	0.0018
			
*Any radiotherapy*			
First hospital visited a radiotherapy centre	1.67	1.32–2.10	<0.0001
Deprivation (Townsend score)	1.00	0.99–1.02	0.7759
Age standardised incidence (per 100 000)	1.00	0.99–1.01	0.9748
			
*Any non-investigate surgery*			
First hospital visited a radiotherapy centre	1.02	0.63–1.64	0.9383
Deprivation (Townsend score)	0.98	0.95–1.01	0.1206
Age standardised incidence (per 100 000)	0.97	0.96–0.99	0.0005
			
*1-year survival*^a^			
First hospital visited a radiotherapy centre	1.18	0.96–1.45	0.1146
Deprivation (Townsend score)	1.01	1.00–1.03	0.0827
Age standardised incidence (per 100 000)	0.99	0.98–1.00	0.0208
			
*3-year survival*^a^			
First hospital visited a radiotherapy centre	1.19	0.99–1.43	0.0674
Deprivation (Townsend score)	1.01	1.00–1.03	0.1320
Age standardised incidence (per 100 000)	0.99	0.98–1.00	0.0276

Odds ratios adjusted for sex, age group, histology and stage of tumour, deprivation, lung cancer incidence, whether first hospital attended was a radiotherapy centre and hospital fitted as random effect.

a Also adjusted for active treatment.

. There were no material changes in the associations of survival with all area and health service characteristics. In the analysis of determinants of chemotherapy, radiotherapy and any treatment, the associations of treatment with the lung cancer incidence rates were sensitive to correction of the error.

The main conclusion of the paper that there is significant geographical variation in the treatment of lung cancer, which may be associated with differential access to oncology services, is still supported.

